# Post-operative complications and ADHD

**DOI:** 10.3389/frcha.2022.1032559

**Published:** 2022-11-01

**Authors:** YooJin Yoon, Matthew Kennis, Elijah W. Hale

**Affiliations:** School of Medicine, University of Colorado Anschutz Medical Campus, Aurora, CO, United States

**Keywords:** ADHD, surgery, complications, peri-surgical, stimulants

## Abstract

ADHD is associated with a number of developmental, emotional, social, academic, and cognitive health risks that can affect the adolescents' quality of life. There have been various guidelines published regarding the management of children with ADHD, however, it has been reported that physicians may not adequately screen for ADHD prior to surgery. To our knowledge, there are no such studies studying post-operative complications and outcome rates in adolescents with ADHD. We conducted a retrospective case-control study through the TriNetX databases. We identified patients with ADHD (ICD-10: F90) undergoing a surgical procedure (CPT: 1003143), and a control group of non-ADHD patients undergoing a surgical procedure. From these two pairs of case-control cohorts we compared outcomes of post-procedural infections, post-procedural shock, and any post-procedural complications. We identified 791,481 matched pairs of subjects undergoing surgery where one of the pair had ADHD and the other did not. Among subjects undergoing a surgical procedure, those with ADHD had a significantly higher risk of post-procedural infection and any post-procedural complication, relative to subjects without ADHD. Furthermore, those with ADHD showed a heightened risk of post-procedural complications in all procedural subcategories. These novel findings suggest that ADHD is a notable factor in surgical care and should be given special consideration by both surgeons and psychiatrists.

## Introduction

Attention-deficit and hyperactivity disorder (ADHD) is one of the most frequent childhood-onset psychiatric conditions and is estimated to have a prevalence between 5 and 10% of school-age children [1]. Symptoms of ADHD affecting quality of life are reported to persist into adulthood in up to 65% of childhood onset cases [2]. Diagnosis of ADHD is based on a persistent pattern of inattention and/or hyperactivity-impulsivity that interferes with functioning or development [3]. ADHD is associated with a number of developmental, emotional, social, academic, and cognitive health risks that can affect the adolescents' quality of life [4]. There have been various guidelines published regarding the management of children with ADHD, however, it has been reported that physicians may not adequately screen for ADHD prior to surgery [5, 6]. To our knowledge, there are no such studies studying post-operative complications and outcome rates in adolescents with ADHD [7, 8].

Common post-operative complications include nausea, vomiting, bleeding from the wound or internally, infections, constipation, and pain [9]. The top five most common types of post-operative complications across all surgical specialties are an unplanned return to the operating room, sepsis, superficial surgical site infection, organ/space surgical site infection, and urinary tract infection [9]. Preventive care is important in the post-operative setting as it reduces pain and provides a marked enhancement of recovery, while also leading to a reduction in the number of hospital days [10].

While some studies have investigated niche surgical fields relevant to the ADHD population, the association of general surgical outcomes amongst patients with ADHD has received less attention [7, 8]. This study aims to gain insight on the relationship between ADHD and the increased risk of post-operative surgical complication rates and outcomes. We hypothesize that patients with ADHD will experience higher rates of post-operative complications at large, rather than exclusively within niche fields.

## Methods

We conducted a retrospective case-control study through the TriNetX database, using International Classification of Diseases, 10^th^ Revision (ICD-10), Current Procedural Terminology (CPT) and Veteran's Affairs (VA) class medication codes [11]. We identified patients with ADHD (ICD-10: F90) undergoing a surgical procedure (CPT:1003143), and a control group of non-ADHD patients undergoing a surgical procedure. As a subgroup analysis, we compared ADHD patients undergoing a surgical procedure and with prescriptions for ADHD stimulants (VA:CN801, VA:CN802) versus a group of ADHD patients undergoing a surgical procedure and without prescriptions for stimulants. From these two pairs of case-control cohorts we compared outcomes of post-procedural infections, post-procedural shock, and any post-procedural complications. The outcome of any post-procedural complication was further subdivided into a post-procedural complication occurring due to one of the following procedural domains: (1) infusion, transfusion, and therapeutic injection; (2) cardiac and vascular prosthetic devices; (3) genitourinary prosthetic devices; (4) orthopedic prosthetic devices; (5) transplanted organs and tissues; (6) reattachment and amputation; (7) other internal prosthetic devices (8) complications of procedures, not classified elsewhere; (9) other complications of surgical care, not elsewhere classified. The time period of the outcome analysis was defined as the outcome of interest occurring at any amount of time following a surgical procedure.

Data was collected between August 8, 2002, and August 8, 2022, as the data were extracted on August 8, 2022. TriNetX, LLC is compliant with the Health Insurance Portability and Accountability Act (HIPAA), the US federal law which protects the privacy and security of healthcare data, and any additional data privacy regulations applicable to the contributing HCO [11]. TriNetX is certified to the ISO 27001:2013 standard and maintains an Information Security Management System (ISMS) to ensure the protection of the healthcare data it has access to and to meet the requirements of the HIPAA Security Rule. Any data displayed on the TriNetX Platform in aggregate form, or any patient level data provided in a data set generated by the TriNetX Platform only contains de-identified data as per the de-identification standard defined in Section §164.514(a) of the HIPAA Privacy Rule [11].

Statistical analysis was performed using the TriNetX software. Comparison between cohorts based on ADHD diagnosis or the use of stimulants was performed with a *t*-test to assess risk differences. Odds ratios were calculated from outcome incidence within each cohort. To prevent confounding variables, we balanced the cohorts on age, sex, and race using propensity score matching with a difference between propensity scores < 0.1. Analysis of each outcome did not exclude patients who had the outcome of interest prior to analysis time period. Significance for this study was set at *p* < 0.05 after adjustment. As this study contained only deidentified aggregate data, the Colorado Multiple Institutional Review Board (COMIRB) designated it as non-human research not in need of approval.

## Results

We identified 791,481 matched pairs of subjects undergoing surgery where one of the pair had ADHD and the other did not. For the subgroup analysis, we identified 337,147 matched pairs of ADHD subjects undergoing surgery, where one of the pair had stimulant prescriptions and the other did not. After cohort balancing according to age, sex, and race, both paired cohorts displayed no significant differences in these demographic characteristics (Table 1).

**Table 1:** Comparison of demographic characteristics after cohort matching between (1) subjects undergoing surgery with ADHD vs. subjects undergoing surgery without ADHD and (2) ADHD subjects without stimulant prescriptions undergoing surgery vs. ADHD subjects with stimulant prescriptions undergoing surgery.

**Variable**	**Surgery with ADHD**	**Surgery without ADHD**	***P*-value**
	***N* = 791,481**	***N* = 791,481**	
Age (years), mean (± SD)	29.9 (±16.4)	29.9 (±16.4)	1
Male Sex (% of Cohort)	423,600 (54%)	423,600 (54%)	1
White (% of Cohort)	590,948 (74%)	590,948 (74%)	1
**Variable**	**Surgery and ADHD without stimulants**	**Surgery and ADHD with stimulants**	***P*** **-value**
	***N*** = **337,147**	***N*** = **337,147**	
Age (years), mean (±SD)	29.5 (±16.6)	29.5 (±16.4)	0.131
Male Sex (% of Cohort)	186,213 (55%)	186,213 (55%)	1
White (% of Cohort)	242,670 (72%)	242,670 (72%)	1

**Figure 1:**
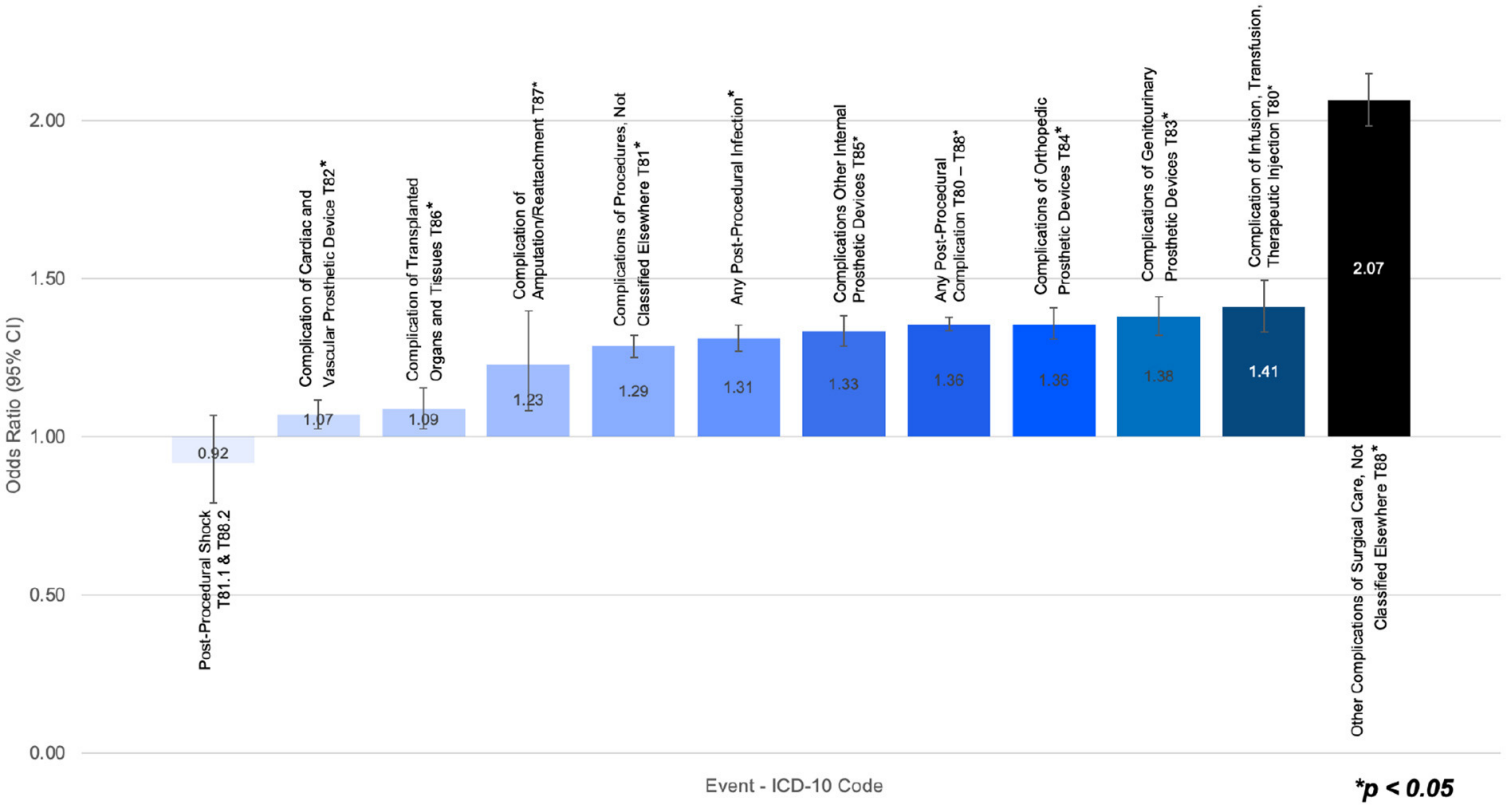
Odds ratio of post-surgical infection, post-surgical shock, or post-surgical complications in subjects undergoing surgery with ADHD vs. subjects undergoing surgery without ADHD.

Among subjects undergoing a surgical procedure, those with ADHD had a significantly higher risk of post-procedural infection and any post-procedural complication, relative to subjects without ADHD. Furthermore, those with ADHD showed a heightened risk of post-procedural complications in all procedural subcategories. The paired cohorts displayed no significant differences in the risk of post-procedural shock (Table 2). Figure 1 presents a graphical representation of the odds ratio between post-procedural outcomes, where a ratio of one indicates the ADHD cohort and the non-ADHD cohort had the same likelihood of event.

**Table 2:** Risk of post-surgical infection, post-surgical shock, or post-surgical complications in subjects undergoing surgery with ADHD vs. subjects undergoing surgery without ADHD.

**Event–ICD10 code**	**Total *N* (excluding patients w/outcome prior to window)**	***N* patients with outcome**	**Risk**	**Odds ratio (95% CI)**	***P* value**
**Any complication T80-88**					
ADHD	791,481	37783	4.77%	1.356558765	0
No ADHD	791,481	28206	3.56%	(1.335, 1.378)	
**Any infection**					
ADHD	791,481	9171	1.16%	1.311698991	0
No ADHD	791,481	7011	0.89%	(1.271, 1.353)	
**Shock T81.1 & T88.2**					
ADHD	791,481	325	0.04%	0.918045444	0.266
No ADHD	791,481	354	0.04%	(0.79, 1.067)	
**Infusion, transfusion, injection T80**					
ADHD	791,481	2721	0.34%	1.410525778	0
No ADHD	791,481	1931	0.24%	(1.331, 1.495)	
**Procedural complications T81**					
ADHD	791,481	12063	1.52%	1.285329931	0
No ADHD	791,481	9417	1.19%	(1.251, 1.321)	
**Vascular T82**					
ADHD	791,481	4301	0.54%	1.069480223	0.002
No ADHD	791,481	4023	0.51%	(1.024, 1.117)	
**Genitourinary T83**					
ADHD	791,481	4786	0.60%	1.380758627	0
No ADHD	791,481	3472	0.44%	(1.322, 1.443)	
**Orthopedic T84**					
ADHD	791,481	6660	0.84%	1.356937212	0
No ADHD	791,481	4919	0.62%	(1.308, 1.408)	
**Other internal devices T85**					
ADHD	791,481	6887	0.87%	1.332429283	0
No ADHD	791,481	5180	0.65%	(1.285, 1.382)	
**Transplants T86**					
ADHD	791,481	2247	0.28%	1.087330659	0.006
No ADHD	791,481	2067	0.26%	(1.024, 1.154)	
**Amputation/Reattachment T87**					
ADHD	791,481	525	0.07%	1.229660534	0.03
No ADHD	791,481	427	0.05%	(1.082, 1.397)	
**Other/Unspecified T88**					
ADHD	791,481	7233	0.91%	2.065147388	0
No ADHD	791,481	3519	0.44%	(1.983, 2.15)	

**Figure 2:**
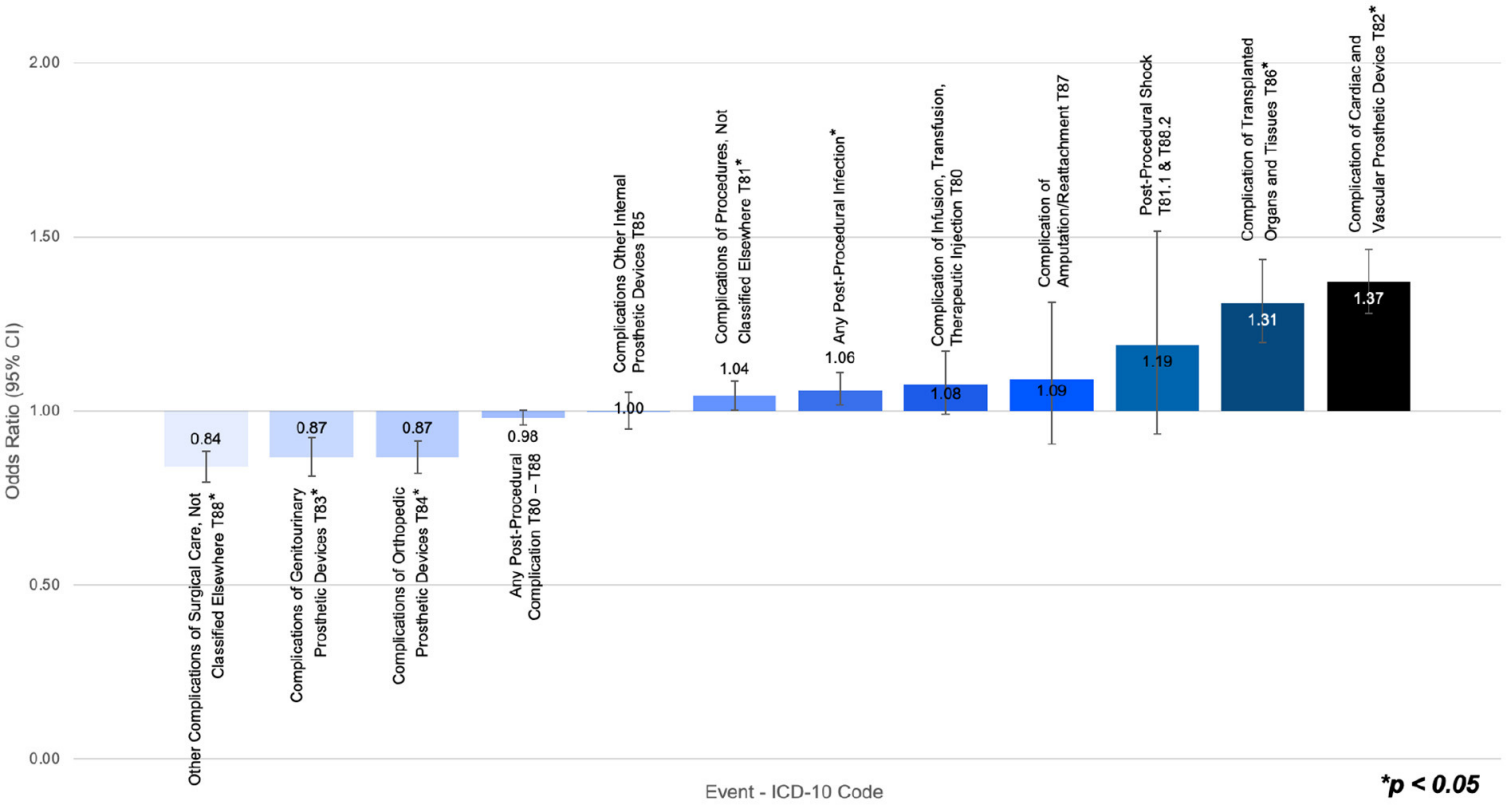
Odds ratio of post-surgical infection, post-surgical shock, or post-surgical complications in ADHD subjects undergoing a surgical procedure without stimulant prescriptions vs. ADHD subjects undergoing a surgical procedure with stimulant prescriptions.

Among ADHD subjects undergoing a surgical procedure, those without stimulant prescriptions had a significantly higher risk of post-procedural infection, relative to ADHD subjects with stimulant prescriptions. The paired cohorts displayed no significant differences in the risk of post-procedural shock and any post-procedural complication (Table 3). However, those without stimulant prescriptions showed an elevated risk of post-procedural complications in six out of the nine procedural subcategories. Figure 2 presents a graphical representation of the odds ratio between post-procedural outcomes, where a ratio of one indicates the ADHD without stimulant prescriptions cohort and the ADHD with stimulant prescriptions cohort had the same likelihood of event.

**Table 3:** Risk of post-surgical infection, post-surgical shock, or post-surgical complications in ADHD subjects without stimulant prescriptions undergoing surgery vs. ADHD subjects with stimulant prescriptions undergoing surgery.

**Event–ICD10 code**	**Total *N* (excluding patients w/outcome prior to window)**	***N* patients with outcome**	**Risk**	**Odds ratio (95% CI)**	***P* value**
**Any complication T80-88**					
No stimulants	337,147	14981	4.44%	0.980395674	0.092
Stimulants	337,147	15267	4.53%	(0.958, 1.003)	
**Any infection**					
No stimulants	337,147	3770	1.12%	1.05965584	0.014
Stimulants	337,147	3560	1.06%	(1.012, 1.11)	
**Shock T81.1 & T88.2**					
No stimulants	337,147	144	0.04%	1.190163866	0.158
Stimulants	337,147	121	0.04%	(0.935, 1.516)	
**Infusion, transfusion, injection T80**					
No stimulants	337,147	1133	0.34%	1.07725582	0.083
Stimulants	337,147	1052	0.31%	(0.99, 1.172)	
**Procedural complications T81**					
No stimulants	337,147	4877	1.45%	1.043163935	0.04
Stimulants	337,147	4678	1.39%	(1.002, 1.086)	
**Vascular T82**					
No stimulants	337,147	2047	0.61%	1.369645787	0
Stimulants	337,147	1497	0.44%	(1.281, 1.464)	
**Genitourinary T83**					
No stimulants	337,147	1730	0.51%	0.866482804	0
Stimulants	337,147	1995	0.59%	(0.812, 0.924)	
**Orthopedic T84**					
No stimulants	337,147	2495	0.74%	0.867449293	0
Stimulants	337,147	2873	0.85%	(0.822, 0.915)	
**Other internal devices T85**					
No stimulants	337,147	2702	0.80%	0.999254379	0.978
Stimulants	337,147	2704	0.80%	(0.947, 1.054)	
**Transplants T86**					
No stimulants	337,147	1079	0.32%	1.31045961	0
Stimulants	337,147	824	0.24%	(1.197, 1.435)	
**Amputation/Reattachment T87**					
No stimulants	337,147	231	0.07%	1.08968409	0.367
Stimulants	337,147	212	0.06%	(0.904, 1.313)	
No stimulants	337,147	2592	0.77%	0.838407818	0
Stimulants	337,147	3087	0.92%	(0.796, 0.884)	

## Discussion

We found significant differences in all but one measured outcome between surgical patients with ADHD compared to those without ADHD. Patients with ADHD experienced a higher rate of all surgical complications except for shock, which did not have a significant difference. Interestingly, these findings remained true for a variety of procedure types, from transplant surgeries, amputations, and cardiac procedures. All outcomes in the study had fewer than 5% absolute risk for both groups, with complications related to amputations as low as 0.05%. Therefore, while any surgical complication may be 1.36 times more likely to occur in patients with ADHD, the equivalent number needed to harm (NNH) is over 80–only 1 in every 80 patients experiences harm based on their ADHD status.

The sub-analysis based on medication usage in ADHD revealed a mix of results. While 5 out of 12 measured outcomes lacked statistical significance, the remaining seven showed that stimulants may have a helpful or detrimental association with complications depending on the type of surgery. This is supported by prior research indicating neurologic protective effects of ADHD medication but does present important novel information [12]. For example, patients with a history of stimulant prescriptions had lower rates of infection, general procedural complications, and complications of vascular or transplant types. In contrast, patients without a history of stimulant prescriptions had lower rates of complications related to genitourinary or orthopedic procedures, as well as lower rates of “other” complications. “Other” complications commonly relate to anesthesia-type complications, such as difficult intubations, malignant hyperthermia, or failed sedation [13]. One possible explanation for this is that stimulant medications can cause a tolerance to certain types of anesthesia, leading to failed sedation [14]. However, as with the overall analysis, the absolute rates of complications remained extremely low. Across all medication types studied, no outcome in the medications sub-analysis had more than a 0.16% difference in absolute risk, which is equivalent to a difference in outcomes for fewer than 1 in every 600 patients.

Despite the low rate overall, our findings do indicate patients with ADHD are significantly more likely to experience post-operative complications, which is a novel finding with potential for improved patient outcomes. Surgical complications are an expensive and at times catastrophic occurrence, particularly for major surgeries such as transplants or reattachments [10]. Further awareness to ADHD could reduce transplant rejection and would increase post-operative outcomes. Awareness of psychiatric conditions in surgical care is not new, and many screening tools and procedural adaptations exist for a variety of surgical procedures [15, 16]. However, prior research has indicated that these tools and adaptations are not commonly used [6]. Furthermore, although children with ADHD are known to be less cooperative regarding surgical care [17], there is no established protocol or adaptation for ADHD in surgical patients. Given the strong associations found in our study, it may be worth developing a specific surgical protocol designed to reduce the detrimental impacts of ADHD in post-operative care.

Our study is the largest to date on ADHD and surgical care. While definitive causation is not present, the size of our study provides substantial confidence that post-operative complications are more likely to occur in patients with ADHD. This association may be attributable to known characteristics of ADHD, such as impulsivity, inattention, and hyperactivity, which seem at odds with the standards of calm and restful recovery in post-operative care [8, 10]. Furthermore, one well-studied difference in ADHD cognitive processing is cost-reward analysis [18]. In surgical care, this may take the place of neglecting proper surgical site management, which can be a painful, tedious, and repetitive process [17]. For a patient with ADHD, the potential of a surgical complication may seem less real and cause them to choose short-term comfort rather than avoiding a potential negative outcome. Surgeons may be able to address the altered cost-reward analysis in this patient population by more strongly emphasizing the importance of preventive care, and by impressing the severity of potential complications on their patients, as these strategies have shown potential in other situations involving neurodevelopmental conditions and preventive care [19, 20]. If this small change in patient care were to reduce post-operative infections in patients with ADHD, it could be integrated into an ADHD-specific post-operative care protocol adaptation. If post-operative infections were reduced even to the level of patients without ADHD, the potential reduction of healthcare costs for patients with ADHD may be in the tens of thousands [21].

While we did eliminate several confounding variables such as age, sex, and race through matched pair analysis, our study is not without limitations. Primarily, the limitations originate from the nature of database studies of ICD codes. As the data is deidentified and aggregated, it is not possible to identify longitudinal trends for individuals. There is also a level of variability in medical records based on provider usage; for example, although a provider may enter stimulants in the medications section of the chart, that does not guarantee they are being taken by the patient at that time. Similarly, it is possible that some surgical complications may have been handled without being specifically diagnosed *via* ICD code. However, this limitation is not entirely negative, as it indicates our study provides a useful lower margin for the rate of these complications. Providers are unlikely to chart a diagnosis that didn't occur, so our findings are a conservative estimate of the true rate. Finally, as was mentioned previously, it is not possible to definitively know the cause behind our noted associations based on the deidentified database information.

As the medical community's understanding of both ADHD and post-operative complications deepens, there is useful information to be gained from further examination of the connections between these two fields. The higher rates of surgical complications in patients with ADHD suggest a unique opportunity for further insight into the cause of complications in the first place. Similarly, the difference in complications between patients on stimulants compared to patients without stimulant prescriptions offers potential for improved patient outcomes if the cause behind the associations is fully investigated. These novel findings suggest that ADHD is a notable factor in surgical care and should be given special consideration by both surgeons and psychiatrists.

## Data availability statement

The original contributions presented in the study are included in the article/supplementary material, further inquiries can be directed to the corresponding author.

## Ethics statement

Ethical review and approval was not required for the study on human participants in accordance with the local legislation and institutional requirements. Written informed consent from the patients was not required to participate in this study in accordance with the national legislation and the institutional requirements.

## Author contributions

MK and EH: had full access to all the data in the study, take full responsibility for the integrity of the data, and the accuracy of the data analysis. EH: served as senior author, with contributions to concept, design, and administrative support. MK: critical revision, data support, figure creation, and manuscript drafting. YY: served as first author, contributed to all aspects of the paper other than data collection, and table/figure creation. All authors contributed to the article and approved the submitted version.

## Conflict of interest

The authors declare that the research was conducted in the absence of any commercial or financial relationships that could be construed as a potential conflict of interest.

## Publisher's note

All claims expressed in this article are solely those of the authors and do not necessarily represent those of their affiliated organizations, or those of the publisher, the editors and the reviewers. Any product that may be evaluated in this article, or claim that may be made by its manufacturer, is not guaranteed or endorsed by the publisher.
